# Langerhans cell histiocytosis in adolescent patients: a single-centre retrospective study

**DOI:** 10.1186/s13023-022-02436-0

**Published:** 2022-07-15

**Authors:** Hua-cong Cai, Jia Chen, Ting Liu, Hao Cai, Ming-hui Duan, Jian Li, Dao-bin Zhou, Xin-xin Cao

**Affiliations:** 1 Department of Hematology, Chinese Academy of Medical Sciences and Peking Union Medical College, Beijing, China; 2 State Key Laboratory of Complex Severe and Rare Diseases, Peking Union Medical College Hospital, Chinese Academy of Medical Sciences & Peking Union Medical College, Beijing, China

**Keywords:** Langerhans cell histiocytosis, Adolescence, Clinical characteristic, Genetic profiling

## Abstract

**Background:**

Langerhans cell histiocytosis (LCH) is a myeloid dendritic cell disorder frequently affecting children more than adults. The presentation of LCH varies with age, however, the clinical characteristics and genetic profiles of adolescent LCH remain elusive. To address the knowledge gap, we performed a single-centre retrospective study of 36 adolescent LCH patients aged between 14 and 17 years at Peking Union Medical College Hospital.

**Results:**

At the time of diagnosis, 10 patients were classified as unifocal single system LCH (27.8%), 2 patients had pulmonary single system LCH (5.6%), 5 patients had multifocal single system LCH with bone involvement (13.9%), and 19 patients had multisystem LCH (52.8%). The most prevalent involvement in multisystem patients was the pituitary gland (78.9%), followed by the bone (42.1%), lung (42.1%), and lymph nodes (42.1%). Eight (42.1%) patients had risk organ involvement. *BRAF*^*N486_P490*^ was detected in 50% of patients who underwent next generation sequencing, and *BRAF*^*V600E*^ was detected in one patient. Chemotherapies were the first line treatment in 24 patients. One patient died and thirteen patients relapsed during the follow-up. The estimated 5-year OS rate and EFS rate were 94.7% and 59.0%, respectively.

**Conclusions:**

In this study, we report a large series of adolescent LCH patients. The clinical characteristics of adolescent LCH patients may be close to adult LCH. Compared with pediatric cases, adolescent LCH tends to have more pituitary lesions and pulmonary involvement, fewer skin and hematopoietic involvement, a higher frequency of BRAF deletion mutation, and a lower frequency of BRAF^V600E^ mutation.

**Supplementary Information:**

The online version contains supplementary material available at 10.1186/s13023-022-02436-0.

## Background

Langerhans cell histiocytosis (LCH) is a rare, clonal disorder derived from CD1a-positive and CD207-positive immature myeloid dendritic cells[1], with a wide range of clinical presentations[2, 3]. Children are more frequently affected than adults. The estimated incidence of LCH in children has been reported to be 4.6 cases per 1 million, while the estimated incidence among adults is 1 to 2 cases per million[4]. The presentation of LCH also varies with age. In a study from a French national cohort of 1478 paediatric patients, single-system and multisystem diseases accounted for approximately half of the patients each[5]. Another study in paediatric patients showed that the median age of diagnosis of patients with risk organ involvement was younger than that of patients without risk organ involvement[6, 7]. Liver and spleen involvement can occur in 10–15% of adult cases[8]. In our previous study, adult patients rarely had haematopoietic system involvement[9]. However, studies and guidelines in children with LCH mainly included patients under 14 years old[10–12], while studies in adults mainly included patients 18 years or older[8]. With relatively low disease frequency, adolescent LCH patients (14–17 years old) have not been reported separately from younger patients in previous studies. There is a lack of information on the clinical features of the adolescent LCH patients cohort.

Recurrent *BRAF*^V600E^ mutations were first discovered in 57% of LCH samples in 2010[13], and alternative activating *MAPK* pathway gene mutations have been discovered since then[14, 15]. In our previous study, unlike that in paediatric patients, *BRAF*^*V600E*^ occurred in only 38.8% of adult LCH patients, while in-frame deletions of exon 12 of the BRAF gene were identified in 25.4% of adult LCH patients[9]. The spectrum of genetic alterations and the impact of these genetic mutations on the clinical presentations of adolescent LCH remains elusive.

To address this knowledge gap, we retrospectively studied the clinical features, treatment approaches, genomic analyses, and outcomes of adolescent LCH patients in our centre over the last twenty years.

## Methods

### Patients

Patients aged between 14 and 17 years at initial onset who were diagnosed with LCH between January 2001 and December 2021 at Peking Union Medical College Hospital (Beijing, China) were included in this retrospective study. Histological findings were consistent with LCH based on the World Health Organization classification[16]. In accordance with the ethical standards of the Declaration of Helsinki, informed consent was obtained from all patients, and the study was approved by the Peking Union Medical College Hospital Ethics Committee.

### Data collection

Data, including baseline clinical features, family history, personal history, physical examination results, biological data, radiological data, treatment data, and survival data, were collected. This series of adolescent patients was compared with 266 adult LCH patients in our centre[9] and 95 paediatric patients in West China Second University Hospital[17] (Sichuan, China) during the same period.

### Genetic analysis and organ involvement

Patients with available samples underwent next-generation sequencing of 183 genes as previously described[18]. The patients were classified according to the number of organs (or systems) involved[8, 19]: SS-s, one lesion within a single system or one organ; SS-p, pulmonary as the single system involvement; SS-m, multiple lesions within one single system; and MS, multiple systems involved. The disease extent was evaluated at the time of the initial diagnosis. Risk organs and their involvement were defined as previously described[9]. MS patients were further classified as patients with risk organ involvement (RO +), and patients without risk organ involvement (RO-).

### Treatment

Most patients with SS-s LCH were treated with local therapies. SS-m and MS LCH patients were mostly treated with systemic treatments. Therapies in this cohort included MA (methotrexate/cytarabine)[20], cytarabine monotherapy (cytarabine 100 mg/m^2^ per day for 5 days), vindesine and prednisone-based chemotherapy[21], cladribine monotherapy (cladribine 5 mg/m^2^ per day for 5 days) and radiation (20 ~ 30 Gy).

Disease status was assessed at three-month intervals during the treatment using the standard evaluations defined by the Histiocyte Society criteria. The patients were classified as follows: complete remission (CR): all signs and symptoms were resolved, all target lesions disappeared, and organ enlargement regressed to normal with no new lesions; partial remission (PR): signs and symptoms were improved, and target lesions regressed by more than 50% with no new lesions; progressive disease (PD): target lesions increased more than 50% and/or new lesions appeared; stable disease (SD): patients did not meet any of the above criteria. The overall response rate (ORR) was defined as the cumulative number of patients with either CR or PR.

### Outcomes

Overall survival (OS) was calculated from the date of the diagnosis of LCH to the date of death or the date of the last contact. Event-free survival (EFS) was calculated from the initiation of treatment for LCH to the first event, and patients who did not have documented events were censored on the date of the last contact. Events were defined as disease reactivation during or after treatment or death from any cause.

### Statistical analysis

The clinical and demographic characteristics of the study participants are summarized using descriptive statistics. All analyses were performed using SPSS statistics version 24.0 (SPSS Inc., Chicago, IL, USA). Fisher’s exact test was used to compare categorical variables, whereas the nonparametric *T* test was used to compare continuous variables between groups. A *P* value < 0.05 was considered significant. OS and EFS were estimated according to Kaplan–Meier survival analysis and compared with the log-rank test. The final follow-up date was December 30, 2021.

## Results

### Demographic data

This cohort included 36 adolescent patients. The baseline characteristics are summarized in Table 1. Overall, 25 patients were male (69.4%), with a male-to-female ratio of 2.3:1. The median age at diagnosis was 16 years (range, 14–17 years). The median time from disease onset to diagnosis was 5.9 months (range 1–144 months). One patient developed central diabetes insipidus when she was 2 years old and was diagnosed with LCH at 14 years old.

**Table 1:** Patient demographics and clinical characteristics

Characteristic	n = 36
Age, years, median (range)	16 (14–17)
*Sex*	
Male, n (%)	25 (69.4)
Female, n (%)	11 (30.6)
*Organ involvement*	
SS-s, n (%)	10 (27.8)
SS-p, n (%)	2 (5.6)
SS-m, n (%)	5 (13.9)
MS, n (%)	19 (52.8)
*SS-s organ involvement (n = 10)*	
Bone, n (%)	4 (40.0)
Pituitary, n (%)	3 (30.0)
Lymph node, n (%)	2 (20.0)
Soft tissue, n (%)	1 (10.0)
*MS organ involvement (n = 19)*	
Pituitary, n (%)	15 (78.9)
Bone, n (%)	8 (42.1)
Lung, n (%)	8 (42.1)
Lymph node, n (%)	8 (42.1)
Liver, n (%)	6 (31.6)
Spleen, n (%)	6 (31.6)
Thyroid, n (%)	5 (26.3)
Skin, n (%)	3 (15.8)
Risk organ, n (%)	8 (42.1)

SS-s, single-system unifocal disease; SS-p, pulmonary as the single system involvement; SS-m, single-system multifocal disease; MS, multisystem disease

### Disease classifications

At the time of diagnosis, 10 patients were classified as having SS-s LCH (27.8%), 2 patients were classified as having SS-p LCH (5.6%), 5 patients were classified as having SS-m LCH with bone involvement (13.9%), and 19 patients were classified as having MS LCH (52.8%). Among the 10 patients with SS-s, 4 had unifocal bone lesions, 3 had isolated pituitary involvement, 2 had isolated lymph node involvement and 1 had other single lesions (soft tissue). The most common organ involved in MS patients was the pituitary gland (78.9%), followed by bone (42.1%), lung (42.1%), lymph nodes (42.1%), liver (31.6%), spleen (31.6%), thyroid (26.3%) and skin (15.8%). Peripheral blood counts were normal in all patients at diagnosis. Eight (42.1%) patients had at least one risk organ involved.

### Family and personal histories

Four patients had family histories, including one lung cancer, one acute leukaemia, one eye tumour, and one brain tumour. One patient was diagnosed with diabetes insipidus. Only one patient in this cohort had a history of smoking and had SS-p LCH first. The patient was required to cease smoking; after 46.8 months, he was reactivated as MS LCH.

### Genomic profiling

Only ten patients had sufficient DNA for next-generation sequencing or retrospective records of BRAF^V600E^ mutation. No significant difference was found in the age, gender, and organ involvement between patients with and without genomic profiles were compared (Additional File 1: Table S1).

At least one somatic mutation was detected in 9 patients (90%). The median number of gene mutations was 2 (range 1–4). *BRAF* or *MAP2K1* alterations were present in 80% of LCH patients, including *BRAF*^V600E^ (10%), *BRAF*^V600D^ (10%), *BRAF*^N486_P490^ (50%), and MAP2K1 (10%). Other somatic mutations included *TP53* (20%), *EGFR* (10%), *MAPK1* (10%), *PTEN* (10%), *NF1* (10%), *RUNX1* (10%) and *SETBP1* (10%) (Table 2).

**Table 2:** Clinical and somatic mutations in Langerhans cell histiocytosis

ID	Sex	Age (years)	Disease classification	Involved organ	Somatic mutations	EFS (months)	Disease status
1	M	17	MS	P,Lu,Li,Sp	*BRAF*^*N486_P490*^	31.4	Stable
2	M	15	MS	P,Lu,Ty,LN	*BRAF*^*N486_P490*^*,EGFR*^*G721S*^	4.0	ReA
3	F	17	MS	P,Lu	*BRAF*^*N486_P490*^*,TP53*^*G206D*^	25.6	Stable
4	F	16	MS	Lu,Li,S,Sp,Ty	*BRAF*^*N486_P490*^	6.0	ReA
5	M	17	SS-m	B	*MAP2K1*^*F53_Q58delinsL*^*,MAPK1*^*I211T*^	27.0	Stable
6	M	14	SS-s	B	*BRAF*^*N486_P490*^	23.6	Stable
7	M	15	SS-m	B	*BRAF*^*V600E*^	16.0	Stable
8	M	17	SS-s	B	*RUNX1*^*R320X*^*,TP53*^*C176Y*^*,SETBP1*^*D868N*^	5.0	Stable
9	M	16	MS	B, Or	*BRAF*^*V600D*^*, PTEN*^*R335X*^*, NF1*^*Q239X*^	7.5	Stable
10	M	17	SS-s	Lu	*Not detected*	46.8	ReA

M, Male; F, Female; MS, multiple system; SS-s, single system single lesion; SS-m, single system multiple lesions; P, Pituitary; Lu, Lung; B, Bone; Li, Liver; S, Skin; LN, lymph nodes; Ty, thyroid; Sp, Spleen; Or, Orbit; ReA, Reactivated

### Initial treatment and outcomes

**Fig. 1:**
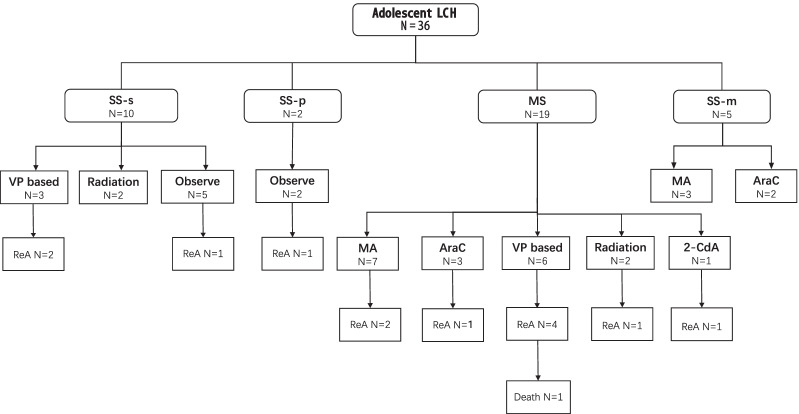
Initial treatment in 36 adolescent LCH patients. A flow diagram demonstrates the initial therapeutic choices of LCH in adolescence, classified by system involvement. LCH: Langerhans cell histiocytosis; SS-s: one lesion within a single system or one organ; SS-p: pulmonary as the single system involvement; SS-m: multiple lesions within one single system; MS: multiple systems involved; VP: vindesine and prednisone; MA: methotrexate/cytarabine; AraC: cytarabine; 2-CdA: cladribine; ReA: reactivated

The initial treatment of the whole cohort is illustrated in a flow diagram in Fig. 1. Treatments of patients with SS-s LCH included systemic VP-based chemotherapy in 3 patients, radiotherapy in 2 patients, and observation in 5 patients. Patients with lung involvement were required to cease smoking and undergo observation. Treatments of patients with SS-m LCH included MA in 3 patients and cytarabine in 2 patients. Treatments for MS included MA in 7 patients, cytarabine in 3 patients, VP-based chemotherapy in 6 patients, radiotherapy in 2 patients, and cladribine monotherapy in one patient.

In total, 9 patients received VP-based chemotherapy as first-line treatment. The ORR was 66.7%, including 2 patients (22.2%) classified as having CR and 4 patients (44.4%) classified as having PR. Two patients (22.2%) were evaluated as SD, and 1 was evaluated as PD. Ten patients received MA as first-line treatment. The ORR was 90%, including 3 patients with CR and 4 patients with PR. One patient was evaluated as having PD. Five patients received cytarabine as first-line treatment. The ORR was 100%, including 2 patients with CR and 2 patients with PR. A response assessment was not yet available for 1 patient. The patient who received cladribine monotherapy as first-line treatment achieved CR.

### Follow-up and Survival

After a median follow-up of 56-months (range 3–181 months), one patient died of disease progression. The estimated 5-year OS rate was 94.7%. No degenerative CNS disease was observed.

**Fig. 2:**
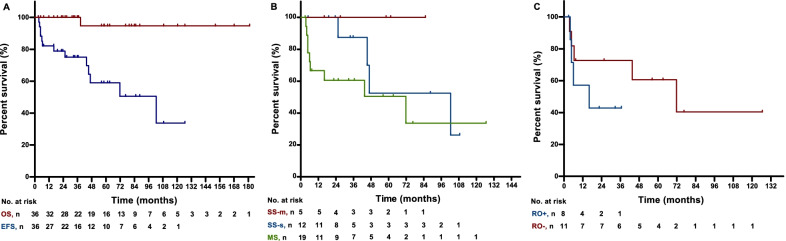
Overall survival and event-free survival of adolescent LCH patients in different subgroups.** A** The OS and EFS of 36 adolescent LCH patients. The estimated 5-year OS rate was 94.7%, and the estimated 5-year EFS rate was 59.0%.** B** The EFS of adolescent LCH patients with different system involvement. The median EFS were 102.8 months, not reached, and 71.8 months for SS-s, SS-m, and MS patients, respectively.** C** The EFS of MS LCH patients with or without risk organ involvement. The median EFS were 16.1 months for patients with risk organ involvement, and 71.8 months for patients without risk organ involvement. LCH: Langerhans cell histiocytosis; OS: overall survival; EFS: event-free survival; SS-s: one lesion within a single system or one organ; SS-p: pulmonary as the single system involvement; SS-m: multiple lesions within one single system; MS: multiple systems involved; RO: risk organ

Thirteen patients had reactivation (Fig. 1). The estimated 5-year EFS rate was 59.0% (Fig. 2A). The median EFS times were 102.8 months, not reached, and 71.8 months for SS-s, SS-m, and MS patients, respectively (*p* = 0.12, Fig. 2B). Among MS patients, the median EFS times were 16.1 months and 71.8 months for RO + patients and RO- patients, respectively (*p* = 0.26, Fig. 2C).

All patients with disease reactivation were reactivated or progressed into MS LCH. The second line therapy included MA in 4 patients, AraC in 3 patients, VP-based chemotherapy in 2 patients, other chemotherapy in 3 patients, and observation in one patient preparing for pregnancy. Three patients had multiple relapses. One patient was treated with VP-based therapy as initial treatment, AraC as the second line therapy, and TCD (thalidomide, cyclophosphamide, dexamethasone) as the third line therapy. One patient was treated with cladribine monotherapy as initial treatment, MA as the second line therapy, and AraC as the third line therapy. One patient died after multiple lines of VP-based therapy and fludarabine chemotherapy.

### Comparative study with children and adult LCH patients

We compared the 36 adolescent patients with 266 adult LCH patients and 95 paediatric patients (Table 3). The difference in male-to-female ratios between adolescent and adult and paediatric LCH patients was not significant (*p* = 0.728 and 0.074, respectively). The disease classification of adolescent LCH patients was quite similar to that of adults and children: MS LCH was the most frequent diagnosis in the three groups of LCH patients (52.8%, 68.4%, and 46.3%, respectively), followed by SS-s LCH (27.8%, 15.0%, and 27.3%) and SS-m LCH (13.9%, 9.8%, and 25.2%).

**Table 3:** Comparison between adult and children LCH

	Adolescent (current study)	Adult (PUMCH)	Children (China)	P1	P2
*Sex*					
Male, (%)	25 (69.4)	177 (66.5)	49 (51.6)	0.728	0.074
Organ involvement					
SS-s, (%)	10 (27.8)	40 (15.0)	26 (27.3)	0.054	1
SS-p, (%)	2 (5.6)	18 (6.8)	0 (0)	1	0.075
SS-m, (%)	5 (13.9)	26 (9.8)	24 (25.2)	0.392	0.154
MS, (%)	19 (52.8)	182 (68.4)	44 (46.3)	0.062	0.542
^*&*^*MS organ involvement*					
Pituitary, (%)	15 (78.9)	112 (61.5)	8 (18.2)	0.134	< 0.001
Bone, (%)	8 (42.1)	127 (69.8)	28 (63.6)	0.015	0.113
Lung, (%)	8 (42.1)	111 (61.0)	17 (38.6)	0.111	0.796
Lymph node, (%)	8 (42.1)	64 (35.2)	17 (38.6)	0.548	0.796
Liver, (%)	6 (31.6)	42 (23.1)	17 (38.6)	0.405	0.593
Spleen, (%)	6 (31.6)	15 (8.2)	9 (20.5)	0.007	0.353
Thyroid, (%)	5 (26.3)	25 (13.7)			
Skin, (%)	3 (15.8)	48 (26.4)	22 (50.0)	0.413	0.011
Haematopoietic system, (%)	0 (0)	0 (0)	9 (20.5)	1	0.047
Risk organ, (%)	8 (42.1)	47 (25.8)	20 (45.5)	0.13	0.806
*Genomic profiling*					
BRAF^V600E^, (%)	1 (10.0)	26 (38.8)	48 (57.1)	0.090	0.006
BRAF deletion, (%)	5 (50.0)	17 (25.4)	2 (2.4)	0.138	< 0.001
MAP2K1, (%)	1 (10.0)	13 (19.4)	1 (1.2)	0.679	0.202
*Outcomes*					
Reactivation rate (%)	*SS-s 33.3%	*SS-s 27.5%		0.696	NA
	SS 23.5%		SS 12.0%	NA	0.249
	SS-m/MS 37.5%	SS-m/MS 45.8%		0.441	NA
	MS 47.4%		MS 15.9%	NA	0.008
EFS (%)	3-y EFS SS-s 87.5% SS-m/MS 69.0%	3-y EFS SS-s 63.3% SS-m/MS 54.7%	5-y EFS 74.6%		

^&^percentage of organ involvement in MS patients

^*^SS-s: including SS-p. NA: p-value calculation was not available because of varied disease classification. P1: *p* values of the chi-square tests between adolescent and adult LCH patients. P2: *p* values of the chi-square tests between adolescent and child LCH patients

In MS LCH patients, lesions in the pituitary gland, bone, lung, and lymph nodes were predominant in both adolescent and adult patients, while paediatric patients were mostly affected by bone and skin lesions. Unlike that in children with LCH, haematopoietic system involvement was absent in adolescents with LCH (20.5% vs. 0%, P = 0.047), while incidences of liver, spleen, and total risk organ involvement were similar between the two groups of patients.

The mutation rates of BRAF^V600E^ were 10%, 38.8%, and 57.1% in adolescent, adult, and paediatric LCH patients, respectively. While BRAF deletion mutations were detected in 50%, 25.4%, and 2.4% of the adolescent, adult, and paediatric LCH patients, respectively. Adolescent LCH patients had a similarly high rate of BRAF deletion mutations to adult patients (50% and 25.4%, *p* = 0.138). Compared to children with LCH, a lower incidence of BRAF^V600E^ (10% vs. 57.1%, P = 0.006) was observed in adolescent LCH patients.

The reactivation rate of adolescent LCH patients resembled that of adult patients (Table 3). Because of varied disease classification in different studies, the reactivation rates were respectively compared in patients diagnosed with SS-s (including SS-p), SS (including SS-s, SS-p, SS-m), SS-m/MS, and MS. A significantly higher reactivation rate was observed in adolescent MS LCH patients compared to children MS LCH patients (47.4% vs 15.9%, P = 0.008). The disease reactivation rates of adolescent SS-s (including SS-p) and SS-m/MS patients were not significant different from adult patients (p-value: 0.696, 0.441). The EFS rates of different groups of patients were also listed in Table 3, but they were not compared because of different follow-up periods or the lack of disease classification details.

## Discussion

We reported adolescent LCH patients in the largest series to date to fill the information gap in LCH patients aged between 14 and 17 years. Adolescent LCH patients in our series displayed a relatively high incidence of lung involvement, a low incidence of haematopoietic system involvement and skin involvement, the absence of neurodegenerative sequelae, and a predominant level of BRAF deletion mutations.

Pulmonary involvement was not unusual in adolescent patients. In a previously published LCH series, paediatric patients displayed a significantly lower incidence of isolated lung involvement than adult LCH patients, as smoking is a key aetiological factor[22–25]. However, our adolescent series displayed a higher tendency of isolated lung involvement of LCH (SS-p) than paediatric patients, although the difference was not significant (5.6% vs. 0%, *p* = 0.075), which is similar to that in adult patients and some previously reported adolescent cases (14–17 years old)[9, 26]. In MS LCH in adolescence, the incidence of pulmonary involvement was not significantly different between adolescents and adults or children. The adolescent MS LCH with pulmonary involvement and without pulmonary involvement had similar clinical outcomes, similar to that of adult MS LCH patients reported previously[27].

A dominant level of pituitary lesions was observed in our results. Diabetes insipidus, as the most common endocrine manifestation of LCH, has been reported in 30–50% of adults[28–32] and 12–28% of children[33, 34]. Nevertheless, one cohort study found that diabetes insipidus occurred more often in children than in adults after a thorough endocrine evaluation[35]. The high prevalence in our results is not surprising because diabetes insipidus can occur at any time before or years after the diagnosis of LCH[36]. Complete evaluation of the endocrine system may also help to screen asymptomatic pituitary stalk enlargement and endocrine dysfunction[35, 36] and increase the incidence of pituitary involvement in LCH.

A significantly lower incidence of skin involvement was revealed in our results for adolescent patients compared to paediatric patients. Previous studies point out that cutaneous LCH is “a great imitator” that has diverse presentations and might contribute to high rates of misdiagnosis[37, 38]. The proportion of LCH skin involvement decreases with age[11, 39], with an incidence of 38–68% in children with LCH[37, 40, 41] and 5–37% in older children or adults with LCH[28, 29, 39]. The incidence of skin involvement in adolescent LCH patients in our results resembles the incidence of cutaneous lesions in adult LCH patients.

Regarding risk organ involvement, the incidence of haematopoietic system involvement was low in adolescent and adult patients. Adolescent LCH patients might have a higher tendency of spleen and liver involvement than adult patients (10–23%)[28, 29, 31], although the difference was not significant because of the limited number of patients. Physicians should pay attention to evaluating risk organ lesions in adolescent LCH patients.

We searched for the presence of MAPK pathway mutations in our series. In addition to the BRAF^V600E^ driver mutation, BRAF deletion was detected in half of the patients who underwent DNA sequencing. The same BRAF^N486_490^ mutation has been reported in 25.4% of adult LCH patients[9], while only a few paediatric patients were found to have BRAF deletions[15]*.* BRAF deletions were commonly seen in adolescent patients with MS LCH and with a poor prognosis, which is similar to the results of previously reported adult LCH patients[9, 18]. This suggests that the genetic and molecular background of adolescents might be closer to that of adults with LCH than that of children with LCH. The results of targeted agent response and molecular studies might be necessary to determine the optimal inhibitors of the MAPK pathway in adolescent LCH patients[15].

The overall treatment plan of adolescent LCH patients resembles first-line therapy choices for adult LCH patients.

After first-line therapy, the estimated 5-year OS rate of adolescent MS LCH patients in our cohort was 94.7%, which is close to the OS of paediatric MS LCH patients in clinical trials[4, 42–44]. With a median follow-up of 56 months, 13 of 36 adolescent patients (36.1%) had disease progression or reactivations in our study. In adolescent MS LCH patients, the 5-year reactivation rate was 36.4% in patients without risk organ involvement and no less than 50% in patients with risk organ involvement. This incidence of reactivation resembles the result of adult MS LCH patients (50–60%)[9, 45]. The median EFS of 16.1 months for adolescent MS LCH patients with risk organ involvement resembles the median EFS previously reported in adult high-risk MS LCH patients [9]. In addition, no neurodegenerative LCH was observed in our adolescent series, which is consistent with the low incidence of CNS sequala previously reported in adult patients (3.8–10%) compared to that in paediatric patients[9, 28, 31, 35]. Unlike the better EFS of paediatric SS-s LCH patients than that of MS patients[5, 45, 46], SS-s patients in our adolescent series had a similar EFS compared to MS patients (102.8 months vs. 71.8 months, *p* = 0.132), which is similar to that of adult patients[9].

Our study has some limitations. Recall and selection biases due to the retrospective nature of the study might affect the accuracy of our results. This is a single-centre study with a limited number of 36 adolescent patients. With a limited number of cases, it was hard to completely avoid the impact of treatment differences on prognosis analysis. When comparing adolescent series with LCH patients of other age groups, geographic features may lead to bias; we mainly analysed the adult cohort previously reported by our centre, which is one of the largest adult LCH cohorts. A multicentre and larger adolescent LCH cohort might be necessary to demonstrate the difference in clinical characteristics and treatment outcomes between adolescents and patients in other age groups.

## Conclusion

We report for the first time a large series of adolescent LCH patients. Our findings should encourage clinicians to be aware of the distinctions and associations between this group of patients and paediatric (< 14 years old) and adult (> 18 years old) LCH patients. Compared with pediatric cases, adolescent LCH tends to have more pituitary lesions, fewer skin and hematopoietic involvement, and a lower frequency of BRAF^V600E^ mutation. Our data suggest that the clinical and genetic characteristics and outcomes of adolescent LCH patients may be close to those of adult LCH patients. However, this hypothesis remains to be confirmed by more adolescent patients in future reports.

## Supplementary Information

**Additional file 1. Table S1.** Comparison between patients with or without genetic profile.

## Data Availability

The datasets used and/or analysed during the current study are available from the corresponding author on reasonable request.

## Abbreviations

LCH: Langerhans cell histiocytosis
MA: Methotrexate/cytarabine
CR: Complete remission
PR: Partial remission
PD: Progressive disease
SD: Stable disease
ORR: Overall response rate
OS: Overall survival
EFS: Event-free survival
SS-s: Single-system unifocal disease
SS-p: Pulmonary as the single system involvement
SS-m: Single-system multifocal disease
MS: Multisystem disease
