# Supplementary material for: Langerhans cell histiocytosis in adolescent patients: a single-centre retrospective study
Source: Orphanet J Rare Dis. 2022 Jul 15;17:268. doi: 10.1186/s13023-022-02436-0 (PMC9288061; doi:10.1186/s13023-022-02436-0)
Supplement: Supplementary file 1 — Additional file 1. Table S1. Comparison between patients with or without genetic profile. [file 13023_2022_2436_MOESM1_ESM.docx]

| **Table S1. Comparison between patients with or without genetic profile** | | | |
| --- | --- | --- | --- |
| **Characteristic** | **with genomic profile (N=10)** | **without genomic profile (N=26)** | **P-value** |
| **Age, years, median (range)** | 16 (14~17) | 16 (14~17) | 0.200 |
| **Sex** |  |  |  |
| Male, n (%) | 8 (80.0) | 17 (65.4) | 0.394 |
| **Organ involvement** |  |  |  |
| SS-s, n (%) | 2 (20.0) | 8 (30.8) | 0.518 |
| SS-p, n (%) | 1 (10.0) | 1 (3.8) | 0.470 |
| SS-m, n (%) | 2 (20.0) | 3 (11.5) | 0.511 |
| MS, n (%) | 5 (50.0) | 14 (53.8) | 0.836 |
| Risk organ, n (%) | 2 (40.0) | 6 (42.9) | 0.913 |

SS-s, single-system unifocal disease; SS-p, pulmonary as the single system involvement; SS-m, single-system multifocal disease; MS, multisystem disease
